# A novel method to rescue and culture duck Astrovirus type 1 in vitro

**DOI:** 10.1186/s12985-019-1218-5

**Published:** 2019-09-05

**Authors:** Ruihua Zhang, Jingjing Lan, Haie Li, Junhao Chen, Yupeng Yang, Shaoli Lin, Zhijing Xie, Shijin Jiang

**Affiliations:** 1 Department of Preventive Veterinary Medicine, College of Veterinary Medicine, Shandong Agricultural University, Taian, 271018 Shandong China; 2 Shandong Provincial Key Laboratory of Animal Biotechnology and Disease Control and Prevention, Taian, 271018 Shandong China

**Keywords:** DAstV-1, DNA-launched infectious clone, Propagation, Trypsin

## Abstract

**Background:**

Reverse genetics systems enable the manipulation of viral genomes and therefore serve as robust reverse genetic tools to study RNA viruses. A DNA-launched rescue system initiates the transcription of viral genomic cDNA from eukaryotic promoter in transfected cells, generating homogenous RNA transcripts in vitro and thus enhancing virus rescue efficiency. As one of the hazardous pathogens to ducklings, the current knowledge of the pathogenesis of duck astrovirus type 1 (DAstV-1) is limited. The construction of a DNA-launched rescue system can help to accelerate the study of the virus pathogenesis. However, there is no report of such a system for DAstV-1.

**Methods:**

In this study, a DNA-launched infectious clone of DAstV-1 was constructed from a cDNA plasmid, which contains a viral cDNA sequence flanked by hammerhead ribozyme (HamRz) and a hepatitis delta virus ribozyme (HdvRz) sequence at both terminals of the viral genome. A silent nucleotide mutation creating a *Bgl* II site in the ORF2 gene was made to distinguish the rescued virus (rDAstV-1) from the parental virus (pDAstV-1). Immunofluorescence assay (IFA) and western blot were conducted for rescued virus identification in duck embryo fibroblast (DEF) cells pre-treated with trypsin. The growth characteristics of rDAstV-1 and pDAstV-1 in DEF cells and the tissue tropism in 2-day-old ducklings of rDAstV-1 and pDAstV-1 were determined.

**Results:**

The infectious DAstV-1 was successfully rescued from baby hamster kidney (BHK-21) cells and could propagate in DEF cells pre-treated with 1 μg/ml trypsin. Upon infection of DEF cells pre-treated with trypsin, DAstV-1 mRNA copies were identified after serial passaging, and the result showed that rDAstV-1 and pDAstV-1 shared similar replication kinetics. Animal experiment showed that the rDAstV-1 had an extensive tissue tropism, and the virus was capable of invading both the central and the peripheral immune organs in infected ducklings.

**Conclusions:**

An improved DNA-launched reverse genetics system for DAstV-1 was firstly constructed. Infectious virus recovered from BHK-21 cells could propagate in DEF cells pre-treated with trypsin. This is the first report of the successful in vitro cultivation of DAstV-1. We believe this valuable experimental system will contribute to the further study of DAstV-1 genome function and pathogenesis.

## Background

Members of the *Astroviridae* family are non-enveloped, positive and single-stranded RNA viruses, typically 28 to 30 nm in diameter. This family is subdivided into two genera with *Mamastrovirus* and *Avastrovirus*, known to infect mammalian and avian species, respectively [1]. In 1975, astrovirus was first identified by electron microscopy as the cause of gastroenteritis in infants [2]. Astroviruses mainly infect the young populations of multiple species, including avian and mammalian species both in terrestrial and aquatic environments [3]. As a potential zoonotic virus, astroviruses spread quickly and are distributed globally, resulting in significant economic losses for the breeding industry and posing a threat to human health [4–6].

As a member of the genus *Avastrovirus* of the family *Astroviridae*, duck astrovirus (DAstV) was first reported in England [7]. Up to now, DAstV has been divided into at least four geno-groups: type 1 (DAstV-1), type 2 (DAstV-2), type 3 (DAstV-3) and the newly found type 4 (DAstV-4) [8, 9]. Astrovirus infection usually causes mild and self-limiting gastroenteritis in most animal species but leads to severe symptoms in poultry, closely related to enteric diseases [10–13], even associated with fatal hepatitis in ducklings [14–17].

The complete genome of DAstV-1 is about 7.7 kb in length and composed of three open reading frames (ORFs) (ORF1a, ORF1b and ORF2) flanked by 5′- and 3′-untranslated regions (UTR) [17]. ORF1a and ORF1b encode the non-structural proteins while ORF2 encodes the capsid proteins [17]. Just like other astroviruses, a ribosomal frameshift motif was found in the ORF1a-ORF1b junction of DAstV-1 [18].

Reverse genetics systems, which enable the rescue of infectious viruses, are critical tools for studying viral life cycles and pathogenesis [19–21]. A DNA-launched rescue system using cellular polymerases II has been developed for manipulation of positive-strand RNA virus genomes [22]. The system initiates the transcription of viral genomic cDNA with eukaryotic promoter in transfected cells, generating homogenous RNA transcripts in vitro and thereby enhancing virus rescue efficiency [23]. The utilization of self-cleaving ribozyme elements can improve the rescue efficiency of DNA-launched infectious cDNA clones for RNA viruses, which greatly promotes the study of virus pathogenesis [24, 25]. However, such a system for DAstV-1 study is still unavailable.

Until now, DAstV-1 has not been successfully cultured in vitro using the traditional infecting methods without trypsin treatment. In this study, using the virulent strain D51 as the parental virus, we firstly constructed a DAstV-1 DNA-launched infectious clone with self-cleaving ribozyme element and rescued DAstV-1 by transfecting this plasmid into baby hamster kidney (BHK-21) cells. The rescued DAstV-1 (rDAstV-1) could be propagated in duck embryonic fibroblasts (DEF) cells pre-treated with trypsin. The ducklings were challenged with the rescued virus, and high viral loads of DAstV-1 were identified in all the different tested organs. The DNA-launched infectious clone constructed in this study will be a valuable tool for further studies of the virulence, genome function, and pathogenic mechanisms of DAstV-1.

## Methods

### Cells, viruses and antibodies

BHK-21 cells were cultured in Dulbecco’s Modified Eagle Medium (DMEM) supplemented with 10% fetal bovine serum (FBS) and were used for the transfection of DNA-launched infectious clones. DEF cells were prepared using 9-day-old duck embryos and cultured in DMEM supplemented with 10% FBS at 37 °C with 5% CO_2_.

The DAstV-1 virulent strain D51 (GenBank accession no. MH712856) was isolated in January 2012 from an outbreak of severe duck viral hepatitis in one-week-old ducklings in Shandong province of China. The mortality rate of the duck flock was more than 30%, and the sick ducklings died quickly with typical hemorrhagic hepatitis. After being propagated in 3-day-old ducklings, the D51 strain was isolated from the livers and used for sequence analysis and full-length genomic cDNA clone construction.

The *ORF2* gene of DAstV-1 D51 strain was subcloned into the *EcoR* I and *Xho* I restriction sites of the pET-32a (+) expression vector. The recombinant plasmid was used to transform *Escherichia coli* (*E. coli*) BL21 (DE3), and the expression of the recombinant ORF2 protein (rORF2) was induced by the addition of isopropyl-β-D-thiogalactoside (IPTG). The anti-DAstV-1 polyclonal antibody (PcAb) was obtained from the mice immunized with the purified rORF2 protein and stored in the Molecular Etiology Laboratory at Shandong Agricultural University. Fluorescein isothiocyanate (FITC)-labeled goat anti-mouse antibody and Horseradish peroxidase (HRP)-conjugated goat anti-mouse antibody were purchased from KPL (MD, USA).

### RNA extraction, reverse transcription polymerase chain reaction (RT-PCR) and sequencing

The parental DAstV-1 (pDAstV-1) was propagated in 3-day-old ducklings, and livers were collected for RNA extraction using TRIzol Reagent (Transgen Biotech, China). The extracted RNA was immediately used for cDNA synthesis using RevertAid™ First Strand cDNA Synthesis Kit (Fermentas, Burlington, Canada) according to the manufacturer’s instruction.

To obtain a full-length sequence of the DAstV-1 D51 strain, ten pairs of specific overlapping primers and two pairs of Race-PCR primers were designed to amplify 12 overlapping PCR fragments covering the complete genome were amplified with Ex Taq polymerase (TaKaRa, Dalian, China) (Table 1). The PCR products were subsequently purified and TA-cloned into pMD18-T vector (TaKaRa, Dalian, China) according to the manufacturer’s protocol. The TA-cloned products were transfected into *E. coli* DH5α competent cells and the positive clones were sequenced by Sangon Biotech Co., Ltd. (Shanghai, China).

**Table 1:** Primers used for complete genome sequencing of D51 strain

Primer		Sequence	Localization (in the polyprotein gene)	Fragment length (bp)
1F		CCGAA(G/A)TGGGCGAGTC	1–918	919
1R		CCAGGTGTCAACAATCATGC		
2F		GATGTTTATGGTGAGTTGTACAC(A/T)G	806–1786	972
2R		GCTAGATGGTATTATGCCTCTTG		
3F		TTGCAGCTCACTCTGGCATA	1567–2231	666
3R		TGATGTCACTCCCTGTGAA(G/C)C		
4F		CTCGCGAAAGAAGTTAAGAGTG	2021–2989	971
4R		CCTTCTTCATCGATGTCATTATCC		
5F		CAGAGAATGCTTGATGAAGG(C/T)	2879–4177	1297
5R		TT(T/A)GGATACGCTGGTGTTGA		
6F		CTTGGACTGTGGAAGCATATACC	3977–4740	763
6R		GTTGAAAACTGCCCTGAAGG		
7F		GTTACCAACTGGAGAAGTCTGTCA	4680–5582	903
7R		TCCAAGTGTGACCACTGTTGTC		
8F		GACAACAGTGGTCACACTTGGA	5561–6619	1082
8R		CCATGTGTGGTGTATATTGTGAT(G/A)		
9F		GAGGTGTTCTGGGCAGTTTCAA	6291–7403	1113
9R		CTCCATCATCATCCTCTCACACTG		
10F		AGTGTGAGAGGATGATGATGGA	7381–7620	240
10R		TGTGACCCACATGGTGATTTC		
3′ Race	P1	AGTGTGAGAGGATGATGATGGA	7381–7751	371
	Race1	GACTCGAGTCGACATCGA(T)_18_		
	P2	ACAGCTGCACTTTCTCATGC	7516–7751	236
	Race2	GACTCGAGTCGACATCGA		

### Reconstruction of the expression vector

To facilitate the subcloning of the viral genome, the plasmid pEGFP-N1 (Clonetech, Mountain View, CA, USA) was reconstructed to make sure only three restriction enzyme sites (sequentially as *Asc* I, *Bam*H I and *Xho* I) existing in the multiple cloning sites (MCS). The generated plasmid, designated pABX, was used as the backbone for the DNA-launched infectious of D51 genome.

### Construction of a full-length cDNA clone

**Fig. 1:**
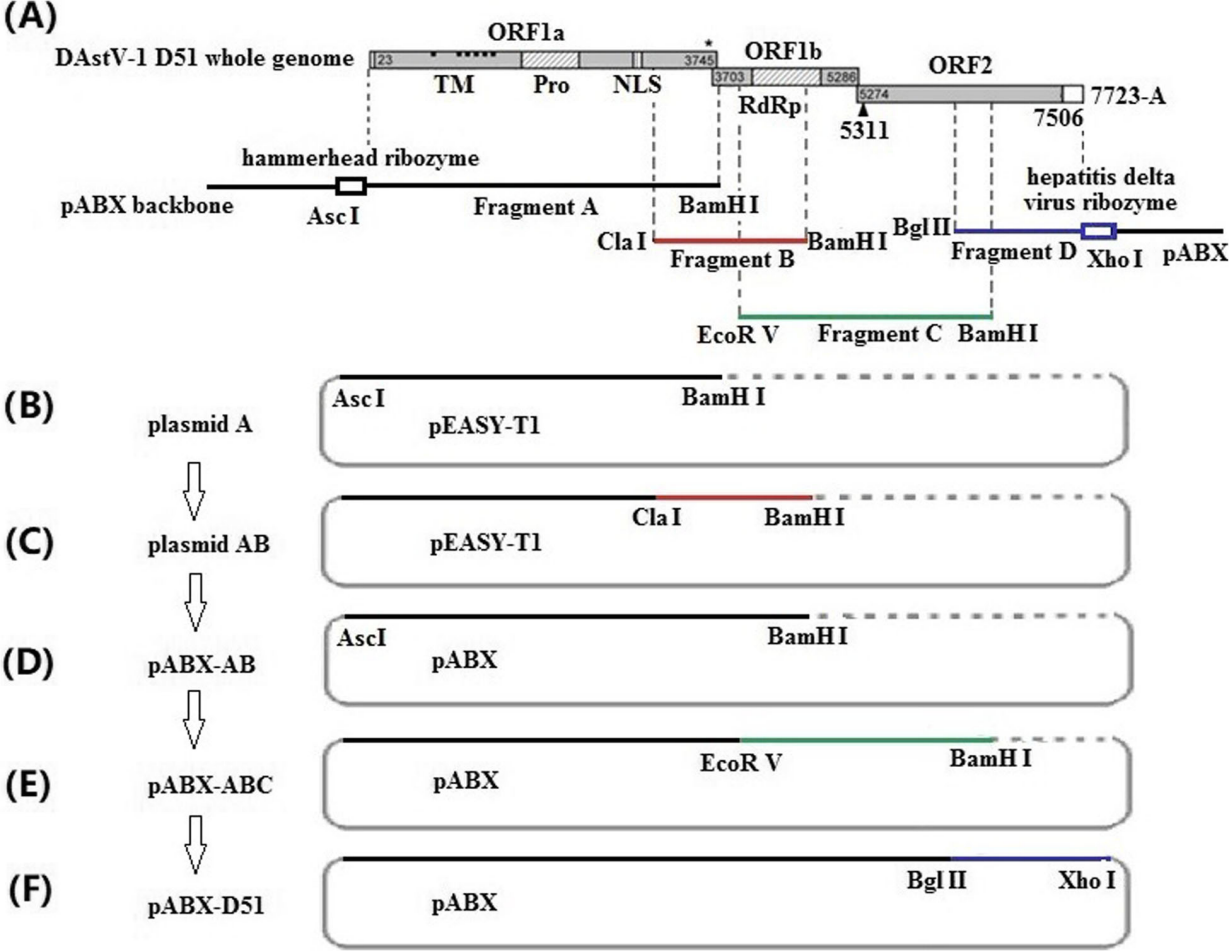
Construction of the DNA-launched infectious cDNA clone of the DAstV-1 D51 strain. (**a**) The organization of the viral genome showing the positions of the unique restriction enzyme site used for cloning. ORF1a, ORF1b and ORF2 indicate the DAstV-1 open reading frames. (**b**, **c**, **d**) Fragments A and B were cloned into pEASY-T1 vector, and then cloned into pABX vector producing pABX-AB. (**e**) Fragment C was fused into pABX-AB releasing pABX-ABC. (**f**) Lastly, fragment D was cloned into pABX-ABC obtaining the complete genome of DAstV-1 D51

A full-length cDNA clone of DAstV-1 D51 was assembled via the subcloning strategy shown in Fig. 1. Briefly, eleven overlapping fragments of the DAstV-1 D51 strain genome was firstly fused into four separate long fragments (FA, FB, FC and FD) using overlap-extension PCR (Fig. 1). After sequencing, FA and FB were then cloned into pEASY-T1 vector (Transgen Biotech, Beijing, China) (named plasmid A, plasmid B), while the FC and FD were cloned into pMD18-T vector (named plasmid C, plasmid D) respectively. A *Bam*H I site was introduced into the 3′ end of the fragment FA, FB and FC by primers HeadRiboF1, aR, bR and cR, respectively. Subsequently, the hammerhead ribozyme (HamRz) gene and restriction enzyme site *Asc* I was fused to the 5′ end of the D51 strain genomic cDNA using PCR as following described. Firstly, plasmid A was used as the template to amplify the first fragment with the primers HeadRiboF2/F1-R. Then, the second-round PCR was performed with the primers HeadRiboF1/F1-R, using the purified first-round PCR product as template. Using the same strategy, the hepatitis delta virus ribozyme (HdvRz) gene and restriction enzyme site *Xho* I were added at the 3′-terminus of the genomic cDNA. Primers used to introduce the ribozyme elements into both termini of the D51 strain genome are listed in Table 2. The plasmids A and B were digested with *Cla* I and *Bam*H I restriction enzymes, and ligated with T4 DNA ligase to produce plasmid AB in vector pEASY-T1 (Fig. 1). The plasmid AB was then digested with *Asc* I and *Bam*H I restriction enzymes and cloned into the vector pABX, producing pABX-AB. Subsequently, the fragment C digested with *Eco*R V and *Bam*H I was inserted into the backbone vector pABX-AB, resulting in pABX-ABC. Finally, the full-length genomic cDNA of DAstV-1 (named pABX-D51) was generated by ligating the pABX-ABC plasmid and fragment D using *Bgl* II and *Xho* I restriction enzymes (Fig. 1).

**Table 2:** Primers used for construction of D51 infectious clone

Primer	Sequences(5′-3′)
HeadRiboF1	TTGGCGCGCCACATCATCTGATGAGTCCGTGAGGACGAAACGGTACCCG (*Asc* I)
HeadRiboF2	CGTGAGGACGAAACGGTACCCGGTACCGTCATCCGAAGTGGGCGAGTCGGGGCCATGGC
F1-R	CCAGGTGTCAACAATCATGC
Long-7R	CCAGAACATTGTCTTTATTCCCTGTAATCTGTCCAAGTGTGACCACTGTTGTC
Long-8F	AGGTGCAGGGTCCCCCTGTCAATGATAAAATGACAACAGTGGTCACACTTGGA
aR	CGCGGATCCTTTGGATACGCTGGTGTTG (*Bam*H I)
bF	CAGAGAATGCTTGATGAAGGC
bR	CGCGGATCCGTTGAAAACTGCCCTGAAGG (*Bam*H I)
cF	CTTGGACTGTGGAAGCATATACC
cR	CGCGGATCCTCATGTGTGGTGTATACTGTGATG (*Bam*H I)
dF	GACAACAGTGGTCACACTTGGA
F12-F	ACAGCTGCACTTTCTCATGC
HpaR1	CCGCTCGAGGTCCCATTCGCCATTACCGAGGGGACGGTCCCCTCGGAATGTTGCCCAGCCG (*Xho* I)
HpaR2	CGGAATGTTGCCCAGCCGGCGCCAGCGAGGAGGCTGGGACCATGCCGGCCTTTT
HpaR3	GGACCATGCCGGCCTTTTTTTTTTTTTTTTTTTTTTTTTTTTTTAATGCCAATTGAA
Marker1	AGCTGGATGTCTGTATTTCGGTTCAACAGCCTCCAGATCTCTCAACAGCGCAC (*Bgl* II)
Marker2	GCTGTTGTGGTGAAGATCAGTGGTGCGCTGTTGAGAGATCTGGAGGCTGTTGAACC (*Bgl* II)

Underlined nucleotides represented the restriction sites

To create a *Bgl* II recognition site, a silent mutation (C to T) at nucleotide 6234 of the DAstV-1 D51 genome was generated by overlap extension PCR with the primers marker 1 and marker 2, which was used as a genetic marker to distinguish the rescued virus from parental virus. The resulting plasmid contained the complete genome of the DAstV-1 D51 strain and was named as pABX-D51. The assembled full-length cDNA clone was sequenced for verification (Sangon, Shanghai, China).

### Transfection and propagation of the rescued virus

BHK-21 cells were seeded in 6-well plates until they reached approximately 70–90% confluency, and then transfected with the recombinant plasmid using the Lipofectamine 3000 reagent (Invitrogen, Carlsbad, CA, USA) according to the manufacturer’s instructions. A total of 2 μg plasmid was mixed with 200 μl of Opti-MEM™ medium and 6 μl Lipofectamine™ Reagent. After being incubated for 20 min at room temperature, the mixture was then added directly to the cell medium for 8 h at 37 °C. A negative control was designed with mock pABX vector transfection.

At 60 h post-transfection (hpt), BHK-21 cells were lysed by three cycles of freezing and thawing, and the cell debris was eliminated by centrifuging at 5000 rpm for 5 min. DEF cells were treated with 1 μg/ml trypsin when approximately 70% confluency for 2 h at 37 °C before infection. After washing with PBS for three times, the supernatant of transfected BHK-21 cells was added to the treated DEF cells (one well to one well) for two-hour’s virus adsorption. After washing three times with PBS, the DEF cells were incubated for 60 h with DMEM containing 2% FBS without trypsin as maintenance media. The supernatant of DAstV-1 was harvested as described above for the further infection of new rounds of DEF cells. The third round of infected DEF cells was harvested for RNA extraction. The DNaseI Digestion kit (OMEGA, GA, USA) was used to remove the residual recombinant plasmid during the RNA extraction assay. The whole genome of the rescued virus was sequenced for verification (Sangon, Shanghai, China).

### Detection of the rescued virus by immunofluorescence assay (IFA)

Cells were examined using IFA every five generations as previous described [26]. At 60 hpt, the transfected BHK-21 cells and the infected DEF cells were washed three times with phosphate-buffered saline (PBS, 8.1 mM Na_2_HPO4, 1.5 mM KH_2_PO_4_, 140 mM NaC1, 3.0 mM KC1, pH 7.2) and then fixed by a mixture of acetone and formaldehyde (1:1) for 20 min at room temperature. After being washed five times with PBS, cells were incubated with anti-DAstV-1 PcAb (dilution of 1:500 with PBS) for 1 h at 37 °C. FITC-labeled goat anti-mouse antibody (dilution of 1:100 with PBS) was used as the secondary antibody for another 1 h at 37 °C. The stained cells were then observed under a fluorescence microscope.

### Western blot assay

The 3rd round of infected DEF cells were lysed using RIPA (Radio immunoprecipitation assay) lysis buffer and boiled in 5 × SDS (Sodium dodecyl sulfate) loading buffer for 10 min and incubated for 5 min on ice. The samples were run on SDS 12%-polyacrylamide gels and transferred onto a polyvinylidene fluoride (PVDF) membrane (ThermoFisher, MA, USA) by standard procedures. The membrane was blocked with 5% skimmed milk in PBS for 1 h, and then incubated with anti-DAstV-1 PcAb (dilution of 1:500 with PBS) in 5% skimmed milk in PBS. After washing three times in PBS, the membrane was incubated in HRP-conjugated goat anti-mouse antibody (dilution of 1:5000) at 37 °C for 1 h. Western blot images were captured using standard enhanced chemiluminescence.

### Analysis of genetic marker of the recombinant and parental viruses

To distinguish recombinant progeny virus from the pDAstV-1, a genetic marker was introduced, a *Bgl* II recognition site, into the cDNA clone via creating a silent mutation as described above. The third passage of the rDAstV-1 and pDAstV-1 infected DEF cells were used for RNA extraction as previously described. The RT-PCR was performed to amplify a 2243-bp fragment (from 5311 to 7753) containing the *Bgl* II recognition site from the cDNA of recombinant and parental virus, using the primers DAstV-1-F (5′-ATG GCT GGT GAG GCC CTT-3′) and DAstV-1-R (5′-CCG CTC GAG GTC CCA TT-3′). The RT-PCR products were digested with *Bgl* II and then identified using electrophoresis in 1% agarose gel.

### Comparison of growth characteristics between the pDAstV-1 and rDAstV-1

The pDAstV-1 of D51 could be propagated in DEF cells pre-treated with trypsin as described above. To compare the growth characteristics of the rDAstV-1 and pDAstV-1, DEF cells were infected with both the viruses at 0.1 MOI (multiplicity of infection). At 24, 36, 48 and 60 h post infection (hpi), cells were collected to analyze the level of DAstV-1 using reverse transcription quantitative PCR. The forward primer 5′-CAT CCA AAC CTC CAA ACA TCT TG-3′ and the reverse primer 5′-CTG TAC CCT CGA TCC TAC TCG G-3′ were used to determine the DAstV-1. A standard plasmid was constructed and serially diluted by ten folds to establish a standard curve. Finally, a regression curve was constructed to plot threshold cycle (Ct) values against the logarithm of the copy number. A quantitative real time polymerase chain reaction (qRT-PCR) was carried out at a total volume of 20 μl, including 12.5 μl of 2 × UltraSYBR Mixture, 0.5 μl forward primer, 0.5 μl reverse primer and 2 μl cDNA template or standard template, with the remainder consisting of DEPC water. The reaction conditions were 95 °C for 10 min, 40 cycles of amplification at 95 °C for 15 s, and 60 °C for 1 min. Non-template control samples were included in each run. Results were analyzed according to the formula produced by the generation of the standard curve and Ct values for each sample. The cells infected with parental viruses and rescued viruses were also harvested for western blot analysis with anti-DAstV-1 PcAb.

### Tissue tropism analysis of the rDAstV-1

Twenty-four 2-day-old healthy ducklings were randomly divided into two groups and then injected intramuscularly with 10^8.24^ copies (0.2 ml) of the rDAstV-1 and same volume of sodium chloride physiological solution, respectively. The ducklings were fed in different incubators following strict biosafety controls, clinical signs and mortality were observed daily. At 24, 36 and 48 hpi, four ducklings from the infected group and control group were randomly selected and their hearts, livers, spleens, kidneys, thymus, bursa of fabricius (BF), and small intestine samples were collected for qRT-PCR analysis. Sequences of the primers were DAstV-1 F (5′-CAT CCA AAC CTC CAA ACA TCT TG-3′) and DAstV-1 R (5′-CTG TAC CCT CGA TCC TAC TCG G-3′). Data are expressed as mean ± SD.

## Results

### Sequence analysis

Eleven fragments were amplified by ten pairs of overlapping primers and two pairs of race-PCR primers. The genomic RNA of DAstV-1 D51 strain was 7753 nt in length with a 31-nt poly(A) tail, including three overlapping ORFs: ORF1a, ORF1b and ORF2, which showed typical astrovirus genome organization (Fig. 1). Homology analysis showed that the D51 strain shared 98.5 and 99.9% nucleotide similarity with the DAstV-1 C-NGB strain (accession no. NC012437) and WF1201 strain (accession no. JX439643), respectively.

### Construction of a plasmid carrying a full-length cDNA of DAstV-1

The full-length cDNA clone of the D51 strain was assembled into the pABX vector with unique restriction enzyme sites in the genome of DAstV-1, and ribozyme sequences were added to produce the exact termini of viral genomic RNA. The plasmid carrying the full-length cDNA was constructed successfully. To distinguish it from the parental virus, the silent mutation at nucleotide 6234 was introduced to create a *Bgl* II site as a molecular marker (Fig. 1).

### Detection of the rescued virus

**Fig. 2:**
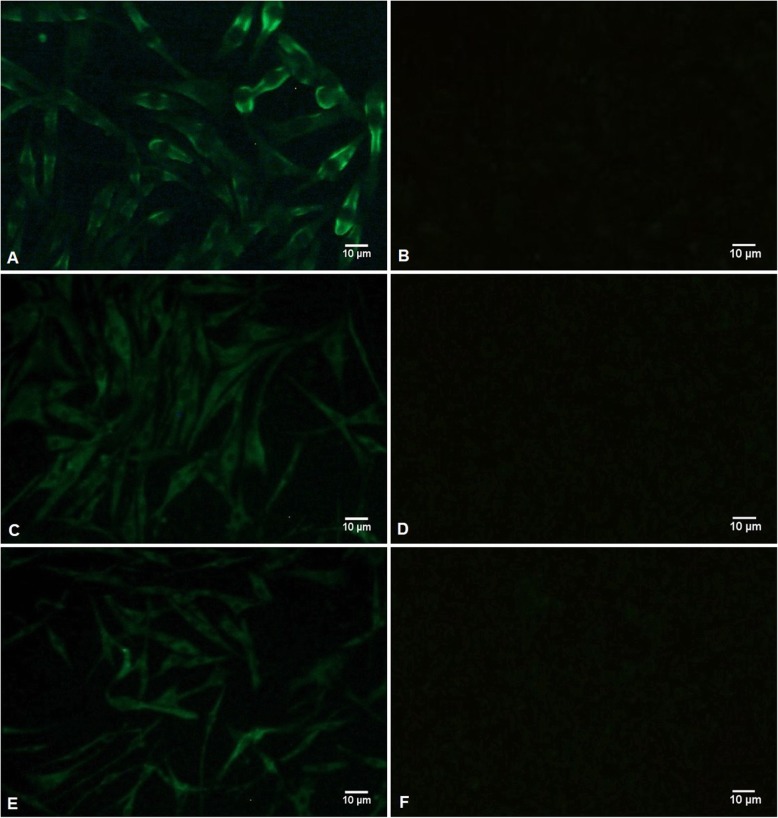
IFA detection of DAstV-1. (**a**) The BHK-21 cells transfected with pABX-D51; (**b**) The BHK-21 cells transfected with pABX; (**c**) The DEF cells infected by the 3rd rescued virus with trypsin treatment; (**d**) The DEF cells infected by the 3rd rescued virus without trypsin treatment; (**e**) The DEF cells infected by the 6th rescued virus with trypsin treatment; (**f**) The DEF cells infected by the 6th rescued virus without trypsin treatment

IFA results showed BHK-21 cells transfected with pABX-D51 could express viral protein (Fig. 2a), while there was no virus antigen in control cells with plasmid pABX transfection (Fig. 2b). After propagation in the DEF cells, the third and sixth passages of the rescued virus were stained positively by IFA (Fig. 2c, e), while the third and sixth passages of the control cells with plasmid pABX transfection were negative (Fig. 2d, f).

**Fig. 3:**
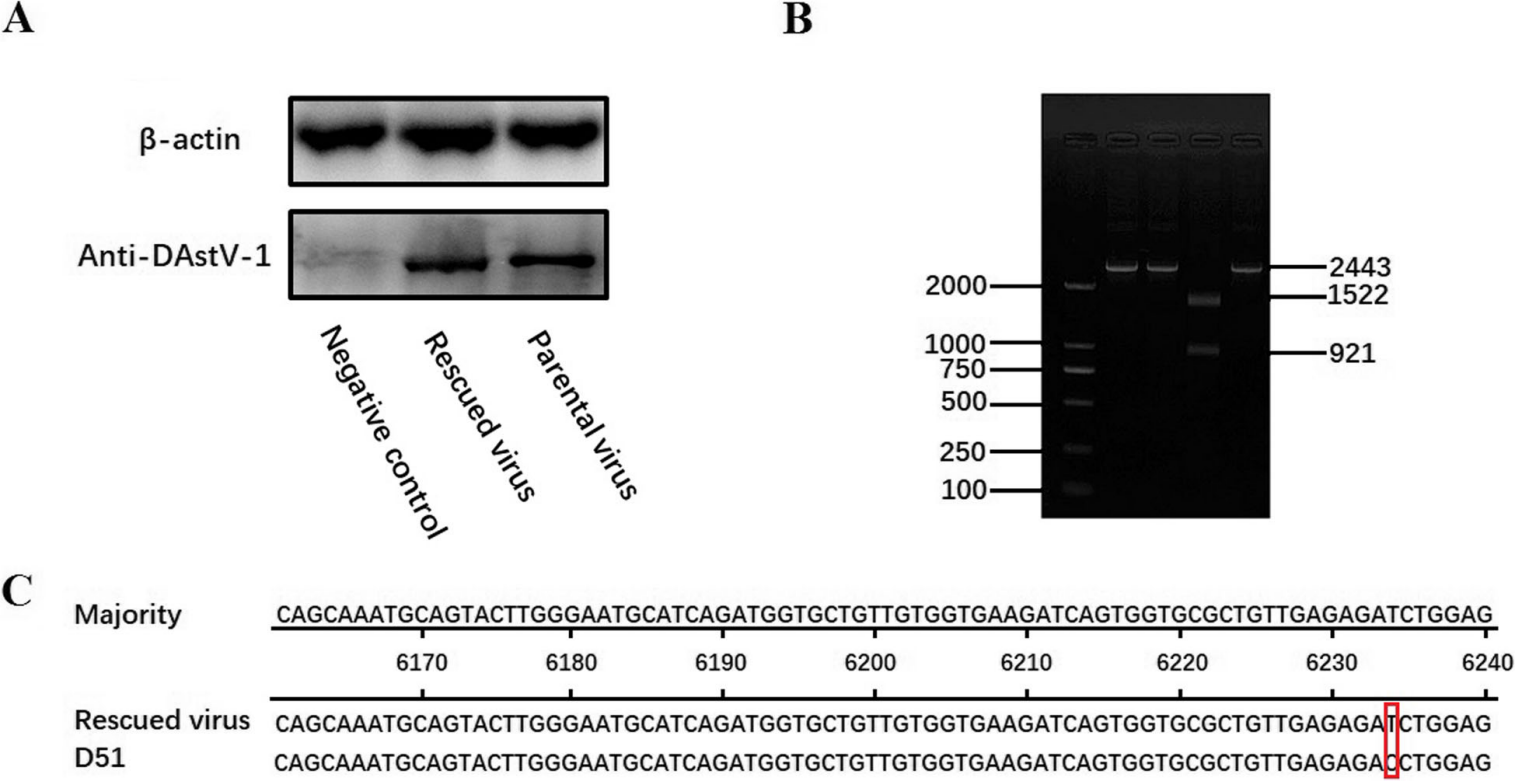
Identification of the rescued virus. (**a**) Western blot analysis of DAstV-1. The DEF cells infected with parental virus and rescued viruses were harvested at 48 hpt with majority anti-DAstV-1 PcAb (dilution of 1:500) and secondary antibody of HRP-conjugated goat anti-mouse antibody (dilution of 1:4000). The mock infected DEF cells were used as negative control. (**b**) Genetic marker of the rescued virus was identified by 1% agarose gel. The *Bgl* II restriction enzyme site was used to distinguish the rescued virus from the parental virus. Using the primers of DAstV-1-F and DAstV-1-R, 2443 bp fragments were identified both in parental and rescued virus. M. DNA Marker DL2000; 1, 2. Fragments amplified from parental and rescued virus; 3. Fragment amplified from rescued virus digested by *Bgl* II with1522 bp fragment and 921 bp fragment; 4. Fragment amplified from the RNA of rescued virus. (**c**) Nucleotide mutation in the rescued virus and the parental virus of D51 strain by sequence alignment

To further confirm that the rescued virus can be obtained from a DNA-launched infection clone, the western blot assay was performed using anti-DAstV-1 PcAb (dilution of 1:500 with PBS). The expression level of the pDAstV-1 and rDAstV-1 at 60 hpt were detected, indicating DAstV-1 could be rescued by transfection with pABX-D51 (Fig. 3a). Meanwhile, the 3rd passage of the rDAstV-1 was sequenced. Compared with the pDAstV-1, there was no base change in the pABX-D51 or rDAstV-1 except for the mutation (from C to T) at 6234 position.

### Identification of the genetic marker in the rDAstV-1

To distinguish the rDAstV-1 from the pDAstV-1, the restriction enzyme *Bgl* II was used to digest the 2443 bp fragment. After being digested with *Bgl* II, the fragment amplified from the rDAstV-1 was digested into two fragments, of 1522 bp and 921 bp, while the fragment amplified from the pDAstV-1 was not cleaved (Fig. 3b). Moreover, the sequence alignment result showed that the rDAstV-1 does have a nucleotide mutation in the position of 6234, consistent with the experimental design (Fig. 3c).

### Analysis of growth characteristics in DEF cells

**Fig. 4:**
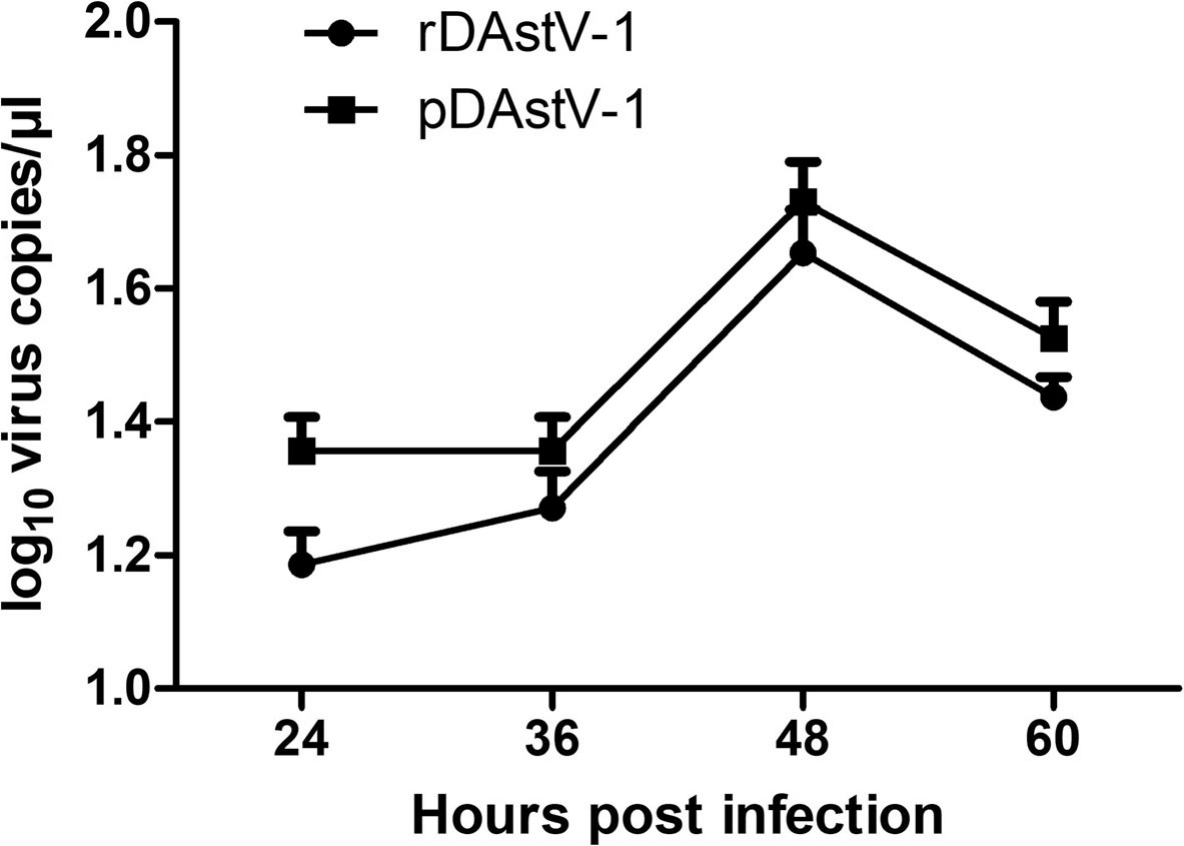
Growth curves of the rescued virus and the parental virus. DEF cells were infected 0.1 MOI of virus recovered from the full-length cDNA clone and the parental virus. At 24, 36, 48 and 60 hpi, the cells were collected and virus copies were determined as described above

To analyze the growth factors of the pDAstV-1 and rDAstV-1, DEF cells were infected with both viruses at 0.1 MOI. The qRT-PCR results revealed that the rDAstV-1 had a similar replication trend to those of the pDAstV-1 (Fig. 4). Both viral RNA copies peaked at 48 hpi, and there was a slight decrease in virus copies after 48 hpi.

### Tissue tropism analysis of the rDAstV-1

Using the qPCR method, the rDAstV-1 was detected in all tested organs (heart, liver, spleen, kidney, thymus, bursa and intestines) of the infected ducklings. In our study, the average viral loads of DAstV-1 in the different organs were 10^6.82^ to10^8.83^ copies/g at 24 hpi, 10^7.39^ to10^7.77^ copies/g at 36 hpi and 10^6.35^ to10^7.02^ copies/g at 48 hpi (Table 3). Notably, the number of viral RNA copies was the highest in the thymus at 24 h (10^8.83^) and the viral load remains high at 48 h (10^6.96^). Except in the spleen, the number of virus copies in all organs was the highest at 24 hpi, and then gradually decreased.

**Table 3:** Average DAstV-1 load in the organs of the infected ducklings (Mean ± SD, log10/g)

Hour post infection	heart	liver	spleen	kidney	thymus	BF	small intestine
24	7.63 ± 0.25	7.51 ± 0.23	6.82 ± 0.14	7.75 ± 0.32	8.83 ± 0.36	8.18 ± 0.33	7.54 ± 0.27
36	7.41 ± 0.12	7.48 ± 0.26	7.77 ± 0.12	7.46 ± 0.25	7.60 ± 0.27	7.52 ± 0.38	7.29 ± 0.13
48	6.99 ± 0.22	6.50 ± 0.31	6.89 ± 0.17	6.71 ± 0.18	6.96 ± 0.33	7.02 ± 0.32	6.35 ± 0.24

## Discussion

Thirty years ago, a list of animal species, including humans, cattle, sheep, cats, dogs, deer, chickens, turkeys, and ducks, had been found susceptible to astrovirus infection [2, 10, 15, 27–31]. Recently, there has been a dramatic increase in astroviruses infected in animal species as the advent of advanced molecular assays and pathogen discovery tools [32–34]. Recombination between different strains of astroviruses aggravated the prevention of astrovirus infection [17, 35].

For RNA viruses, the HamRz and HdvRz as self-cleaving RNA ribozyme elements had been introduced into DNA-launched infectious cDNA clones to successfully improve the rescue efficiency [25, 26]. In this study, the two self-cleaving elements were added at both termini of the viral genomic cDNA to generate a DNA-launched infectious clone of DAstV-1. Both In vitro and in vivo experiments revealed that the virus could be rescued with high efficiency. Since human astrovirus (HAstV) infectious clone was successfully constructed in 1977 [36], many cell lines have been used for virus propagation, such as HEK-293 cells, Caco-2 cells, Huh7.5.1 cells and BHK-21 cells [37]. Ernesto Méndez et al. had reported that the processing of the HAstV Yuc8 polyprotein capsid precursor from a 70-kDa protein into viral particles was dependent on trypsin treatment [38]. As a member of avian astroviruses (AAstVs), avian nephritis virus (ANV) had been successfully rescued from transfected chicken kidney cells with the infectious RNA genome transcribed in vitro from a genomic-length cDNA clone [39]. Differing from previously described mammalian astroviruses [40–42], trypsin was not required for ANV growth in tissue culture [39]. Trypsin can enhance the infectivity of AAstVs through facilitating the maturity of the capsid protein [43]. Here, we added culture medium containing 1 μg/ml trypsin before virus infection to facilitate viral adsorption. Whether trypsin acts the same role in DAstV-1 capsid precursor processing as human astrovirus needs to be further studied.

Regarding DAstV-1, both the parental virus and rescued virus could be cultured in DEF cells (Fig. 3a). The growth curve revealed that the rDAstV-1 has similar growth kinetics with the pDAstV-1 (Fig. 4). However, the mRNA copy number of the rDAstV-1 was consistently lower than that of the pDAstV-1 due to unknown reason.

Our previous research had revealed that duck hepatitis A virus (DHAV) type 1 (DHAV-1) and type 3 (DHAV-3) were detected with high viral loads in all the tested organs (liver, spleen, thymus, pancreas, brain, thymus, kidney and BF) of clinical infected Cherry Valley ducklings [44]. In this study, high viral loads of DAstV-1 was firstly identified in all the different tested organs (liver, spleen, kidney, thymus, BF, heart and small intestines) of the infected ducklings, indicating that DAstV-1 also had extensive tissue tropism. The high viral loads of in the immune organs indicated that DAstV-1 could invade both central and peripheral immune organs, which was similar to DHAV [44]. Overall, there was an evident decline of DAstV-1 loads in all the tissues after 36 hpi (Table 3). At 24 hpi, DAstV-1 was detected with high viral loads in all of the tested organs and the viral loads in thymus and BF were significantly higher than those of other organs, indicating DAstV-1 could target the ducklings’ immune organs. Additionally, the viral load change in central immune organs (thymus and BF) and peripheral immune organs (spleen) indicated the immune activation and suppression of viral replication upon DAstV-1 infection.

## Conclusions

In conclusions, an improved DNA-launched reverse genetics system for DAstV-1 was firstly constructed. The infectious virus recovered from BHK-21 cells could propagate in DEF cells pre-treated with trypsin. This is the first report of the successful in vitro cultivation of DAstV-1. The rDAstV-1 shared similar growth characteristics in DEF cells with the pDAstV-1, and the rDAstV-1 had extensive tissue tropism. The study provides a valuable experimental system to investigate the genome function and pathogenesis of DAstV-1.

## Data Availability

All data generated or analyzed during this study are included in this published article.

## Abbreviations

AAstVs: Avian astroviruses
ANV: Avian nephritis virus
BF: Bursa of fabricius
BHK: Baby Hamster Syrian Kidney
Ct: Threshold cycle
DAstV-1: Duck astrovirus type 1
DEF: Duck embryo fibroblast
DHAV: Duck hepatitis A virus
DMEM: Dulbecco’s Modified Eagle Medium
*E. coli*: *Escherichia coli*
FBS: Fetal Bovine Serum
FITC: Fluorescein isothiocyanate
HamRz: Hammerhead ribozyme
HAstV: Human astrovirus
HdvRz: Hepatitis delta virus ribozyme
hpi: Hours post infection
hpt: Hours post transfection
HRP: Horseradish Peroxidase
IFA: Immunofluorescence assay
IPTG: Isopropyl-β-D-thiogalactoside
MCS: Multiple cloning sites
MOI: Multiplicity of infection
ORF: Open reading frame
PBS: Phosphate-buffered saline
PcAb: Polyclonal antibody
pDAstV-1: Parental DAstV-1
PVDF: Polyvinylidene fluoride
qRT-PCR: Quantitative real time polymerase chain reaction
rDAstV-1: Rescued DAstV-1
RIPA: Radio immunoprecipitation assay
rORF2: Recombinant ORF2 protein
RT-PCR: Reverse transcription polymerase chain reaction
SDS: Sodium dodecyl sulfate
UTR: Untranslated regions

## References

[1] Walter JE, Mitchell DK (2003). Astrovirus infection in children. Curr Opin Infect Dis.

[2] Madeley CR, Cosgrove BP (1975). Letter: 28 nm particles in faeces in infantile gastroenteritis. Lancet..

[3] De Benedictis P, Schultz-Cherry S, Burnham A, Cattoli G (2011). Astrovirus infections in humans and animals - molecular biology, genetic diversity, and interspecies transmissions. Infect Genet Evol.

[4] Karlsson EA, Small CT, Freiden P, Feeroz MM, Matsen FA (2015). Non-human Primates Harbor diverse mammalian and avian Astroviruses including those associated with human infections. PLoS Pathog.

[5] Meliopoulos VA, Kayali G, Burnham A, Oshansky CM, Thomas PG (2014). Detection of antibodies against Turkey astrovirus in humans. PLoS One.

[6] Rivera R, Nollens HH, Venn-Watson S, Gulland FM, Wellehan JF (2010). Characterization of phylogenetically diverse astroviruses of marine mammals. J Gen Virol.

[7] Asplin FD (1965). Duck hepatitis. Vet Rec..

[8] Liu N, Wang F, Shi J, Zheng L, Wang X (2014). Molecular characterization of a duck hepatitis virus 3-like astrovirus. Vet Microbiol.

[9] Liao Q, Liu N, Wang X, Wang F, Zhang D (2015). Genetic characterization of a novel astrovirus in Pekin ducks. Infect Genet Evol.

[10] McNulty MS, Curran WL, McFerran JB (1980). Detection of astroviruses in Turkey faeces by direct electron microscopy. Vet Rec..

[11] Cattoli G, Toffan A, De Battisti C, Salviato A, Terregino C (2005). Astroviruses found in the intestinal contents of Guinea fowl suffering from enteritis. Vet Rec..

[12] Pantin-Jackwood MJ, Spackman E, Woolcock PR (2006). Molecular characterization and typing of chicken and Turkey astroviruses circulating in the United States: implications for diagnostics. Avian Dis.

[13] Spackman E, Day JM, Pantin-Jackwood MJ (2010). Astrovirus, reovirus, and rotavirus concomitant infection causes decreased weight gain in broad-breasted white poults. Avian Dis.

[14] Asplin FD (1965). Duck hepatitis: vaccination against two serological types. Vet Rec..

[15] Gough RE, Collins MS, Borland E, Keymer LF (1984). Astrovirus-like particles associated with hepatitis in ducklings. Vet Rec.

[16] Todd D, Smyth VJ, Ball NW, Donnelly BM, Wylie M (2009). Identification of chicken enterovirus-like viruses, duck hepatitis virus type 2 and duck hepatitis virus type 3 as astroviruses. Avian Pathol.

[17] Fu Y, Pan M, Wang X, Xu Y, Xie X (2009). Complete sequence of a duck astrovirus associated with fatal hepatitis in ducklings. J Gen Virol..

[18] Chen L, Xu Q, Zhang R, Li J, Xie Z (2012). Complete genome sequence of a duck astrovirus discovered in eastern China. J Virol.

[19] Aubry F, Nougairede A, Gould EA, de Lamballerie X (2015). Flavivirus reverse genetic systems, construction techniques and applications: a historical perspective. Antivir Res.

[20] Neumann G, Kawaoka Y (2004). Reverse genetics systems for the generation of segmented negative-sense RNA viruses entirely from cloned cDNA. Curr Top Microbiol Immunol.

[21] Yang CC, Hu HS, Wu RH, Wu SH, Lee SJ (2014). A novel dengue virus inhibitor, BP13944, discovered by high-throughput screening with dengue virus replicon cells selects for resistance in the viral NS2B/NS3 protease. Antimicrob Agents Chemother.

[22] Boyer JC, Haenni AL (1994). Infectious transcripts and cDNA clones of RNA viruses. Virology..

[23] Huang YW, Fang Y, Meng XJ (2009). Identification and characterization of a porcine monocytic cell line supporting porcine reproductive and respiratory syndrome virus (PRRSV) replication and progeny virion production by using an improved DNA-launched PRRSV reverse genetics system. Virus Res.

[24] Martin A, Staeheli P, Schneider U (2006). RNA polymerase II-controlled expression of antigenomic RNA enhances the rescue efficacies of two different members of the Mononegavirales independently of the site of viral genome replication. J Virol.

[25] Kato T, Matsumura T, Heller T, Saito S, Sapp RK (2007). Production of infectious hepatitis C virus of various genotypes in cell cultures. J Virol.

[26] Chen J, Zhang R, Lin S, Li P, Lan J (2017). Construction and characterization of an improved DNA-launched infectious clone of duck hepatitis a virus type 1. Virol J.

[27] Woode GN, Bridger JC (1978). Isolation of small viruses resembling astroviruses and caliciviruses from acute enteritis of calves. J Clin Microbiol.

[28] Bridger JC (1980). Detection by electron microscopy of caliciviruses, astroviruses and rotavirus-like particles in the feces of piglets with diarrhoea. Vet Rec..

[29] Williams FP (1980). Astrovirus-like, coronavirus-like, and parvovirus-like particles detected in the diarrheal stools of beagle pups. Arch Virol.

[30] Tzipori S, Menzies JD, Gray EW (1981). Detection of astrovirus in the faeces of red deer. Vet Rec..

[31] Harbour D., Ashley C., Williams P., Gruffydd-Jones T. (1987). Natural and experimental astrovirus infection of cats. Veterinary Record.

[32] Yuan X, Meng K, Zhang Y, Yu Z, Ai W (2018). Genome analysis of newly emerging goose-origin nephrotic astrovirus in China reveals it belongs to a novel genetically distinct astrovirus. Infect Genet Evol.

[33] Salamunova S, Jackova A, Mandelik R, Novotny J, Vlasakova M (2018). Molecular detection of enteric viruses and the genetic characterization of porcine astroviruses and sapoviruses in domestic pigs from Slovakian farms. BMC Vet Res.

[34] Yi S, Niu J, Wang H, Dong G, Guo Y (2018). Molecular characterization of feline astrovirus in domestic cats from Northeast China. PLoS One.

[35] Pantin-Jackwood MJ, Spackman E, Woolcock PR (2006). Phylogenetic analysis of Turkey astroviruses reveals evidence of recombination. Virus Genes.

[36] Geigenmuller U, Ginzton NH, Matsui SM (1997). Construction of a genome-length cDNA clone for human astrovirus serotype 1 and synthesis of infectious RNA transcripts. J Virol.

[37] Velazquez-Moctezuma R, Banos-Lara Mdel R, Acevedo Y, Mendez E (2012). Alternative cell lines to improve the rescue of infectious human astrovirus from a cDNA clone. J Virol Methods.

[38] Mendez E, Fernandez-Luna T, Lopez S, Mendez-Toss M, Arias CF (2002). Proteolytic processing of a serotype 8 human astrovirus ORF2 polyprotein. J Virol.

[39] Imada T, Yamaguchi S, Mase M, Tsukamoto K, Kubo M (2000). Avian nephritis virus (ANV) as a new member of the family Astroviridae and construction of infectious ANV cDNA. J Virol.

[40] Woode GN, Gourley NE, Pohlenz JF, Liebler EM, Mathews SL (1985). Serotypes of bovine astrovirus. J Clin Microbiol.

[41] Willcocks MM, Ashton N, Kurtz JB, Cubitt WD, Carter MJ (1994). Cell culture adaptation of astrovirus involves a deletion. J Virol.

[42] Carter MJ, Willcocks MM (1996). The molecular biology of astroviruses. Arch Virol Suppl.

[43] Bass DM, Qiu S (2000). Proteolytic processing of the astrovirus capsid. J Virol.

[44] Lin SL, Cong RC, Zhang RH, Chen JH, Xia LL (2016). Circulation and in vivo distribution of duck hepatitis a virus types 1 and 3 in infected ducklings. Arch Virol.

